# An integrated convolutional neural network for classifying small pulmonary solid nodules

**DOI:** 10.3389/fnins.2023.1152222

**Published:** 2023-06-02

**Authors:** Mengqing Mei, Zhiwei Ye, Yunfei Zha

**Affiliations:** ^1^School of Computer Science, Hubei University of Technology, Wuhan, China; ^2^Department of Radiology, Renmin Hospital of Wuhan University, Wuhan, China

**Keywords:** medical image analysis, neural networks, classification, pulmonary solid nodules, feature extraction

## Abstract

Achieving accurate classification of benign and malignant pulmonary nodules is essential for treating some diseases. However, traditional typing methods have difficulty obtaining satisfactory results on small pulmonary solid nodules, mainly caused by two aspects: (1) noise interference from other tissue information; (2) missing features of small nodules caused by downsampling in traditional convolutional neural networks. To solve these problems, this paper proposes a new typing method to improve the diagnosis rate of small pulmonary solid nodules in CT images. Specifically, first, we introduce the Otsu thresholding algorithm to preprocess the data and filter the interference information. Then, to acquire more small nodule features, we add parallel radiomics to the 3D convolutional neural network. Radiomics can extract a large number of quantitative features from medical images. Finally, the classifier generated more accurate results by the visual and radiomic features. In the experiments, we tested the proposed method on multiple data sets, and the proposed method outperformed other methods in the small pulmonary solid nodule classification task. In addition, various groups of ablation experiments demonstrated that the Otsu thresholding algorithm and radiomics are helpful for the judgment of small nodules and proved that the Otsu thresholding algorithm is more flexible than the manual thresholding algorithm.

## 1. Introduction

Pulmonary nodule classification is an important task that judges the benignity and malignancy of pulmonary nodules by computer techniques. Deep learning methods based on convolutional neural networks are the most common methods for pulmonary nodule classification, which can be divided into 2D CNN based methods (Setio et al., 2016; Sori et al., 2019) and 3D CNN based methods (Shi et al., 2021; Tsai and Peng, 2022). In general, the 2D CNN based methods for pulmonary nodule classification have three steps: first, the 3D CT images are sliced; then, the features are extracted by 2D CNN; finally, the extracted features are input to the classifier to obtain the results. Shen et al. (2017) constructed an end-to-end architecture, which used neural networks for feature extraction instead of complex nodule segmentation and manual fabrication, and achieved better classification accuracy.

The pulmonary nodule images are three-dimensional, and extracting two-dimensional slices with only one view of the image could easily cause information deficiency. Therefore, some studies have proposed multi-view slicing methods. Xie et al. (2018) proposed to learn the features of 3D pulmonary nodules by 9 fixed views, different views are learned using different sub-models. Al-Shabi et al. (2019) proposed a local-global neural network, which uses the residual module with the convolution kernel size of 3 × 3 to extract local features, and global features are extracted by self-attention layers, the combination of both features achieves better classification results. Considering the three-dimensional characteristics of 3D CT images, many works based on 3D CNNs have appeared in recent years. Kang et al. (2017) proposed a 3D multi-view convolutional neural network to better utilize 3D contextual information and extracted more discriminative features. Liu et al. (2021) proposed a contextual attention network (CA-Met), CA-Met extracts the nodules and surrounding tissues features by contextual attention and then fuses the two features into the classifier for prediction. Zhang et al. (2018) introduced 3D dilated convolution into the base model, which helps the model retain more image information and acquire multi-scale features, leading to more accurate nodule classification results. Huang et al. (2022) proposed a novel neural network based on self-supervised learning. This network learns labeled and unlabeled data to overcome the problem of insufficient labeled samples and eliminates noisy information interference by data preprocessing. Although 3D CNN achieves great classification results, it faces the problems of large computation and complex network structure. Therefore, the pulmonary nodule classification still needs further research to play a greater role in the medical career.

## 2. A 3D residual convolutional neural network

**Figure 1:**
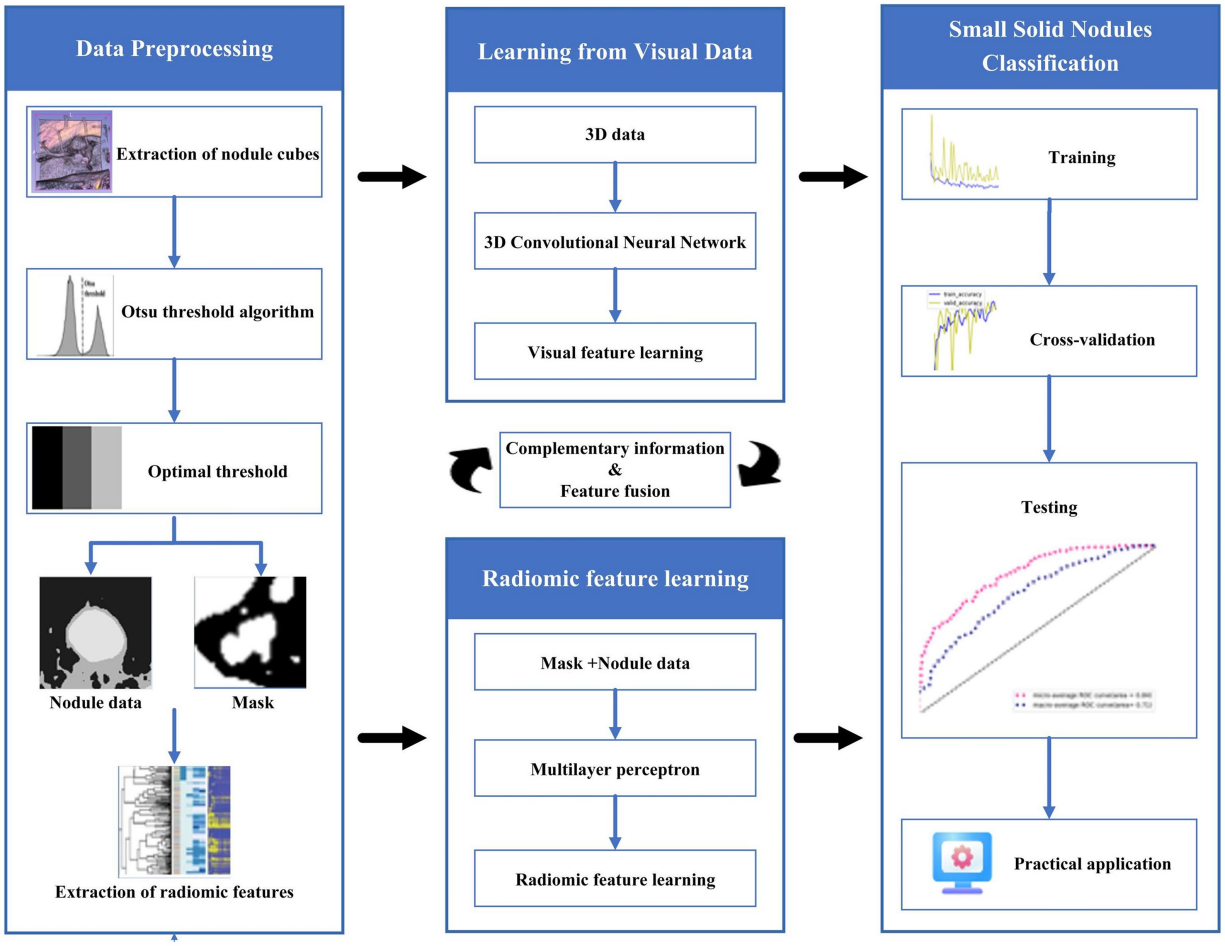
Flowchart of the proposed algorithm.

Providing accurate classification of small pulmonary solid nodules in CT images is significant. For this reason, this paper proposes a classification method for small pulmonary solid nodules. Specifically, we use the Otsu thresholding algorithm to reduce the noise interference from pulmonary tissues around the nodules. The proposed method extracts two modal features through a 3D residual convolutional neural network and radiomics. Then we fuse the visual and radiomic features into the classifier, which helps the model better predict benign and malignant nodules. As shown in Figure 1, the complete framework of the classification method consists of three parts: (1) data preprocessing based on the Otsu thresholding algorithm; (2) the interactive learning based on two modal features of three-dimensional residual convolutional neural network and radiomics; (3) small solid nodules classification.

### 2.1. Data preprocessing

This paper uses the Otsu threshold algorithm for multi-threshold segmentation preprocessing of medical CT images. This algorithm divides the images into three classes, C1, C2, and C3, by two thresholds, K1 and K2, and then obtains the optimal threshold by the maximum inter-class variance. The inter-class variance is formulated as follows:


(1) $$\sigma^{2}=\sum_{k=1}^{3}P_{k}{\left(m_{k}-m_{G}\right)}^{2}$$

where *k* is the corresponding class. $P_{k}$ denotes the probability of class *k*, m refers to the mean gray value of the *k*th class and the formula is as follows:


(2) $$P_{k}=\sum_{i\in C_{k}}p_{i}$$

where $p_{i}$ is the probability of the gray level being *i*. The probability of different classes can be written as:


(3) $$P_{1}=\sum_{i=0}^{K_{1}}p_{i},P_{2}=\sum_{i=K_{1}+1}^{K_{2}}p_{i},P_{3}=\sum_{i=K_{2}+1}^{255}p_{i}$$

The mean gray value of the *k*th class can be defined as:


(4) $$m_{k}=\frac{1}{P_{k}}\sum_{i\in C_{k}}ip_{i}$$

The mean gray value of three classes can be formulated as:


(5) $$m_{1}=\frac{1}{P_{1}}\sum_{i=0}^{K_{1}}ip_{i},\;\;\;m_{2}=\frac{1}{P_{2}}\sum_{i=K_{1}+1}^{K_{2}}ip_{i},\;\;m_{3}=\frac{1}{P_{3}}\sum_{i=K_{2}+1}^{255}ip_{i}$$

The mean gray value of the entire image is calculated as:


(6) $$m_{G}=\sum_{i=0}^{255}ip_{i}$$

Finally, two optimal thresholds $K_{1}^{*}$ and $K_{2}^{*}$ are obtained by maximizing $\sigma^{2}\left(K_{1},K_{2}\right)$, the formula is as follows:


(7) $$\sigma^{2}\left(K_{1}^{*},K_{2}^{*}\right)=\underset{0<K_{1}<K_{2}<255}{\max}\sigma^{2}\left(K_{1},K_{2}\right)$$

If the maximum value of inter-class variance is not unique, the corresponding optimal thresholds $K_{1}^{*}$ and $K_{2}^{*}$ are averaged to obtain the final threshold. The Otsu algorithm is described as follows:

**Table:** 

**Algorithm:** Otsu algorithm
**Input**: grayscale images generated from medical CT images **Output**: optimal thresholds $K_{1}^{*}$ and $K_{2}^{*}$ calculate the normalized histogram and the probability $p_{i}$ that the gray level is *i* (*i* = 0, 1, …, 255) from the input image; calculate the mean gray value $m_{G}=\sum_{i=0}^{255}ip_{i}$ of the entire image; For *K1* = 1:253 For *K2* = *K1* + 1:254 calculate the probability of three classes $P_{1}=\sum_{i=0}^{K_{1}}p_{i},P_{2}=\sum_{i=K_{1}+1}^{K_{2}}p_{i},P_{3}=\sum_{i=K_{2}+1}^{255}p_{i};$ calculate the mean gray value $m{\;}_{1}=\frac{1}{P_{1}}\sum_{i=0}^{K_{1}}ip_{i},m_{\;2}=\frac{1}{P_{2}}\sum_{i=K_{1}+1}^{K_{2}}ip_{i},m_{\;3}=\frac{1}{P_{3}}\sum_{i=K_{2}+1}^{255}ip_{i}$; calculate the inter-class variance $\sigma^{2}\left(K_{1},K_{2}\right)=\sum_{k=1}^{3}P_{k}{\left(m_{k}-m_{G}\right)}^{2}$; End End obtain two optimal thresholds $K_{1}^{*}$ and $K_{2}^{*}$ by (7); If $size\left(K_{1}^{*},1\right)>1$ $K_{1}^{*}=mean\left(K_{1}^{*}\right)$; End If $size\left(K_{2}^{*},1\right)>1$ $K_{2}^{*}=mean\left(K_{2}^{*}\right)$; End

**Figure 2:**
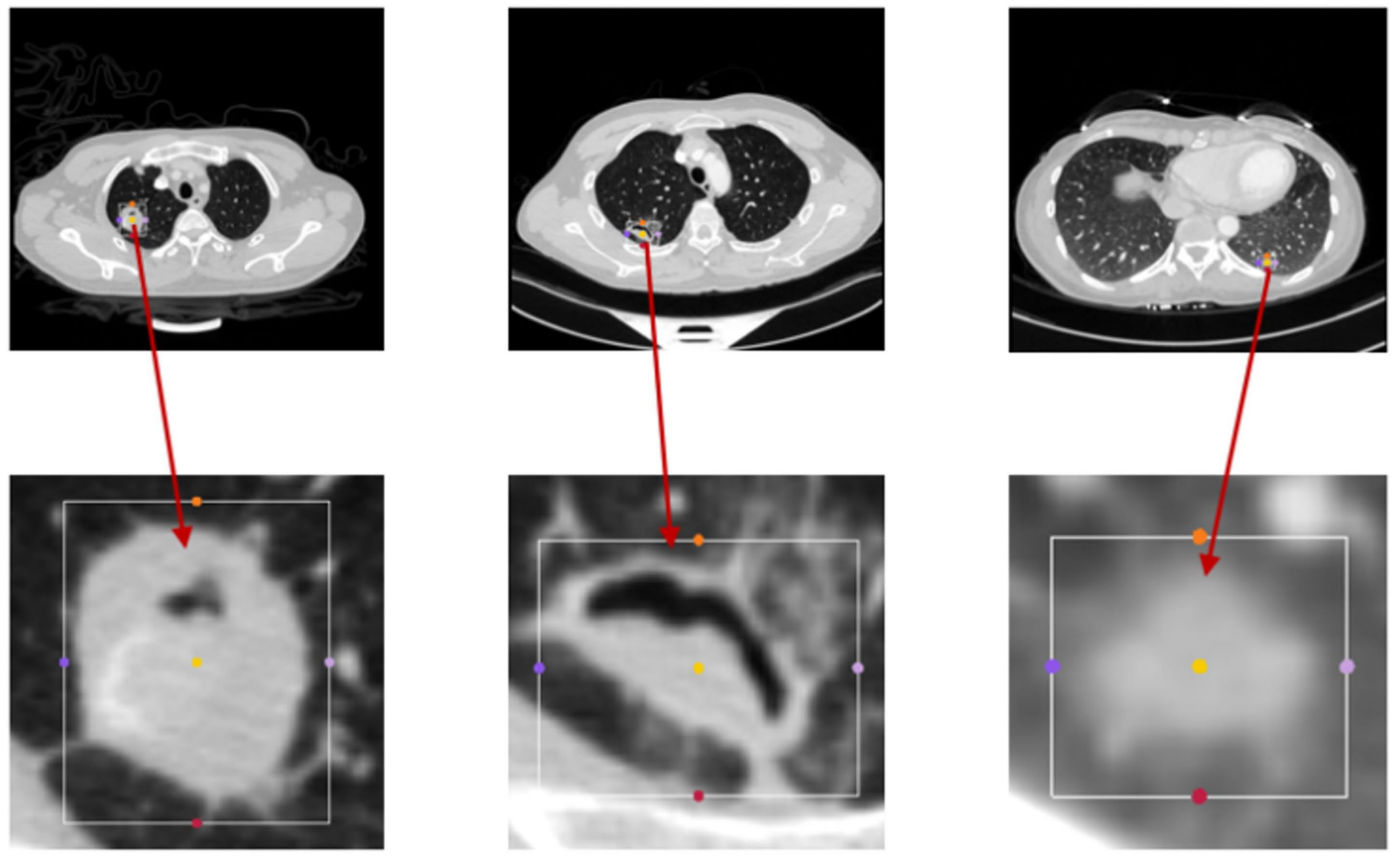
The figure displays the bounding boxes of nodular lesions.

**Figure 3:**
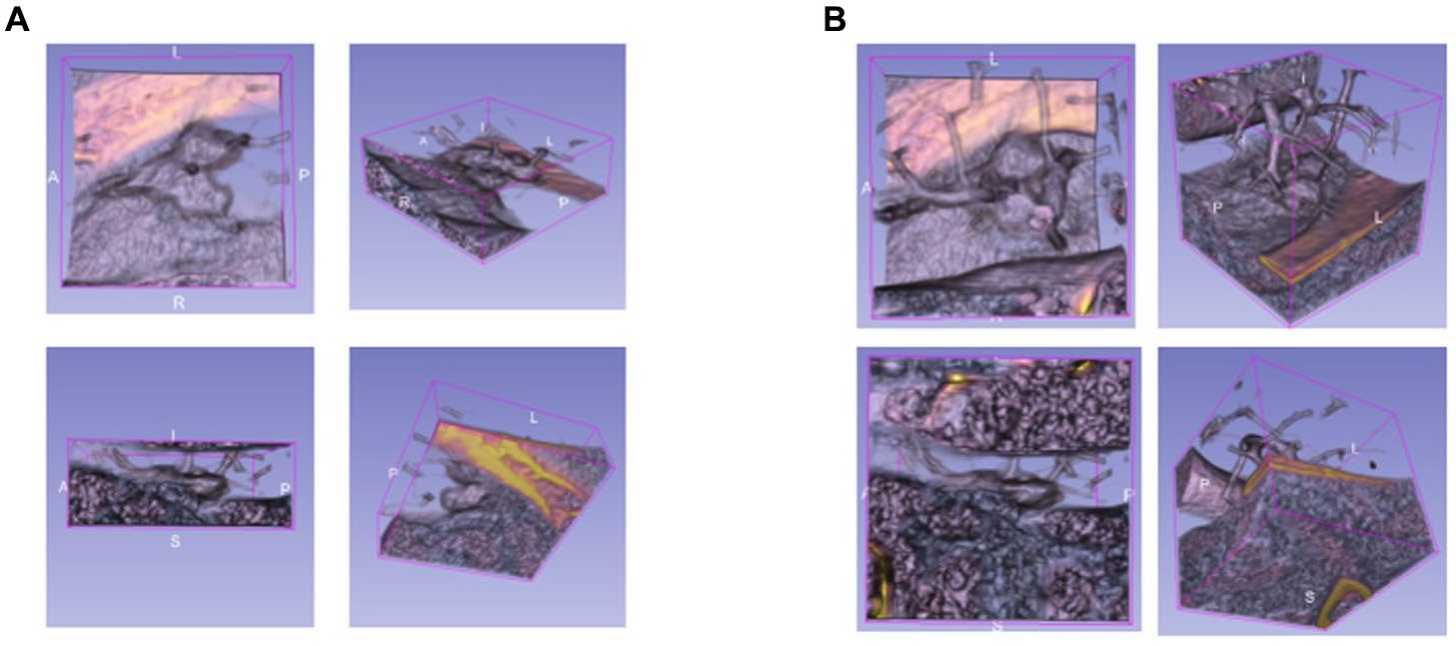
The example of the pulmonary nodules in three dimensions, **(A)** rectangular, **(B)** cubic.

**Figure 4:**
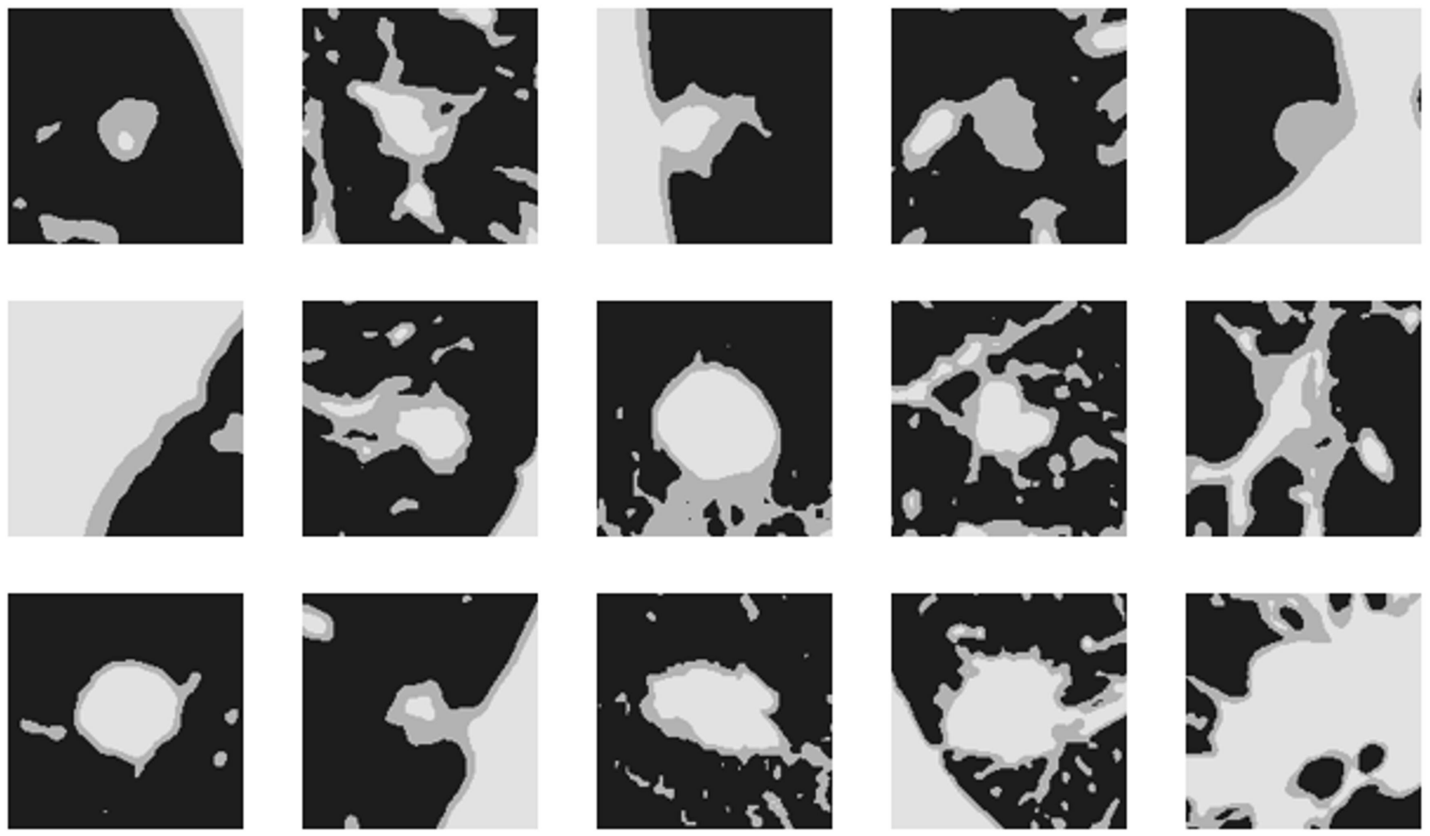
The results of the Otsu thresholding algorithm on CT data.

The bounding boxes of nodular lesions are mainly labeled according to the nodule location information provided by professional radiologists, and the examples are shown in Figure 2. The labeled information is a rectangle composed of six-coordinate points in three-dimensional space, and the six-coordinate points are denoted as $x_{\max?}x_{\min?}\;y_{\max?}y_{\min?}z_{\max?}z_{\min}$. Due to the input requirement of the convolutional neural network is a cube, and the resampling will change the nodule shape, this paper selects the maximum value of *H*, *W*, and *D* as the side length of the cube and uses the padding operation to ensure the integrity of the nodule information, the pairs in three-dimensional space are shown in Figure 3. In addition, considering the interference factors such as blood vessels, air bubbles, and lung lobes, this paper extracts the nodule proper and its edge within a smaller error range by the Otsu thresholding algorithm. Specifically, a three-band thresholding classification method is used in this section, and the last two bands are selected as the retained information. As shown in Figure 4, the black areas indicate the parts that will be ignored, and the gray and white areas are the information that will be retained.

**Figure 5:**
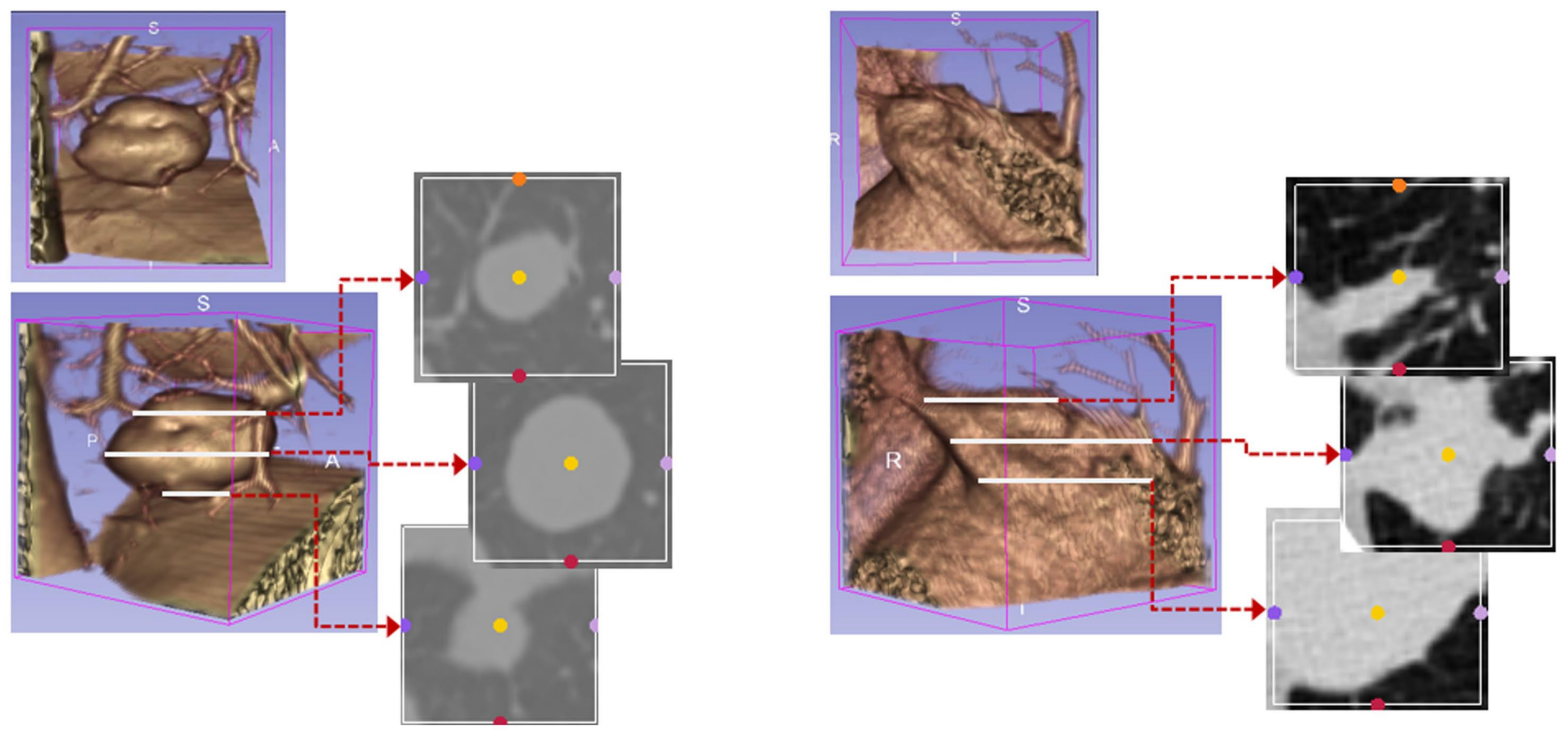
The 3D information of small pulmonary nodules and their corresponding 2D slices.

In common medical image processing, three-dimensional data are treated as *N* two-dimensional slices, computed by two-dimensional convolution kernels to obtain feature maps. These methods suffer from the problem of missing correlations between different slices, leading to inconsistency in the conclusions. In the small solid pulmonary nodule classification task, the shape of the same small pulmonary solid nodule varies greatly between different slices, so it is not easy to achieve the expected results by training with the traditional scheme. Therefore, this paper utilizes the cube containing the whole nodule information for training. The examples of two-dimensional slice information are shown in Figure 5.

**Figure 6:**
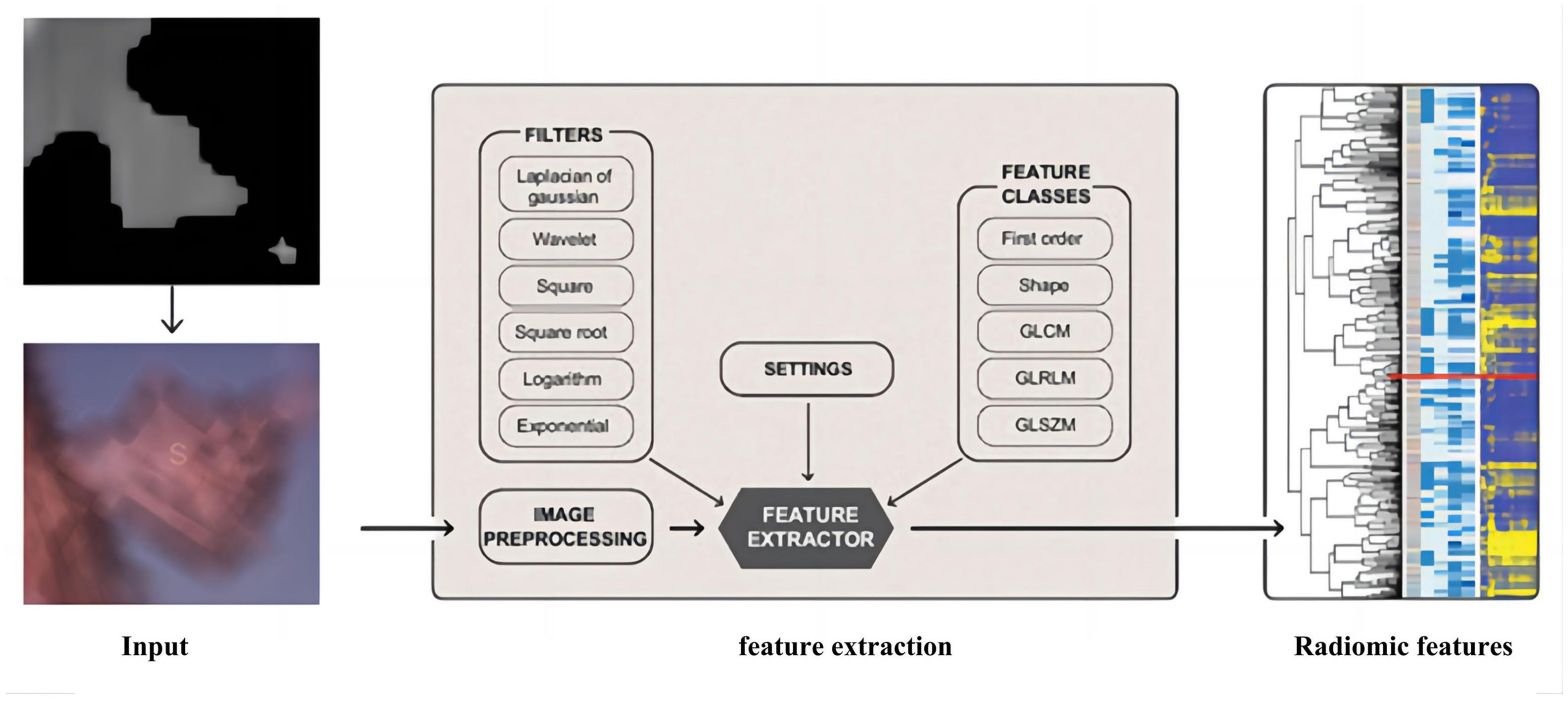
The extraction process of radiomics.

This paper uses an algorithm based on PyRadiomics (a radiomic features extraction package developed by the Harvard Medical School team) to extract radiomic features of nodules. The various features extracted include the following classes: First Order Statistics has 19 features, which mainly include the magnitude of voxel values in the cube, the maximum, minimum and the range of the grayscale value of the lesion area; Shaped-Based (3D), which includes 16 features, such as the voxel volume, surface area, sphericity and surface area to volume ratio; Shaped-Based (2D) have 10 features, mainly including mesh surface, pixel surface, and perimeter to surface ratio; Gray Level Co-occurence Matrix have 24 features, mainly including autocorrelation, joint average, and cluster shade; gray level run length matrix have 16 features, mainly including short and long run emphasis, gray level non-uniformity and run length non-uniformity; gray level size zone matrix have 16 features, especially including small and large area emphasis; Neighbouring Gray Tone Difference Matrix have 16 features, including coarseness, contrast, busyness, complexity and strength; Gray Level Dependence Matrix have 14 features, mainly including small and large dependence emphasis, gray level variance, dependence entropy. In addition, this paper uses the results from the Otsu algorithm as the input mask to ensure that the information obtained from radiomics is accurate, and the extraction process is shown in Figure 6.

Few images and unbalanced class distribution are common problems in medical image processing. This paper uses a data enhancement algorithm based on MONAI to alleviate these problems, which can strengthen the neural network’s generalization. The data enhancement approaches used for the nodular cube include random 3D image rotation, random 3D image flip, and random affine transformation; those approaches will not act on the radiomic features. Considering the non-uniform classification of benign and malignant nodules in the training dataset, the lesser class (benign) will get more enhancement. The nodules will randomly flip along an axis with the probability of 70% benign and 40% malignant. Then, they will be affine transformed with the probability of 70% benign and 40% malignant. Finally, they are randomly rotated with the same probability (the maximum rotation angle for benign is 35° and for malignant is 30°).

### 2.2. Fusion learning of 3D residual convolutional neural networks and radiomics

**Figure 7:**
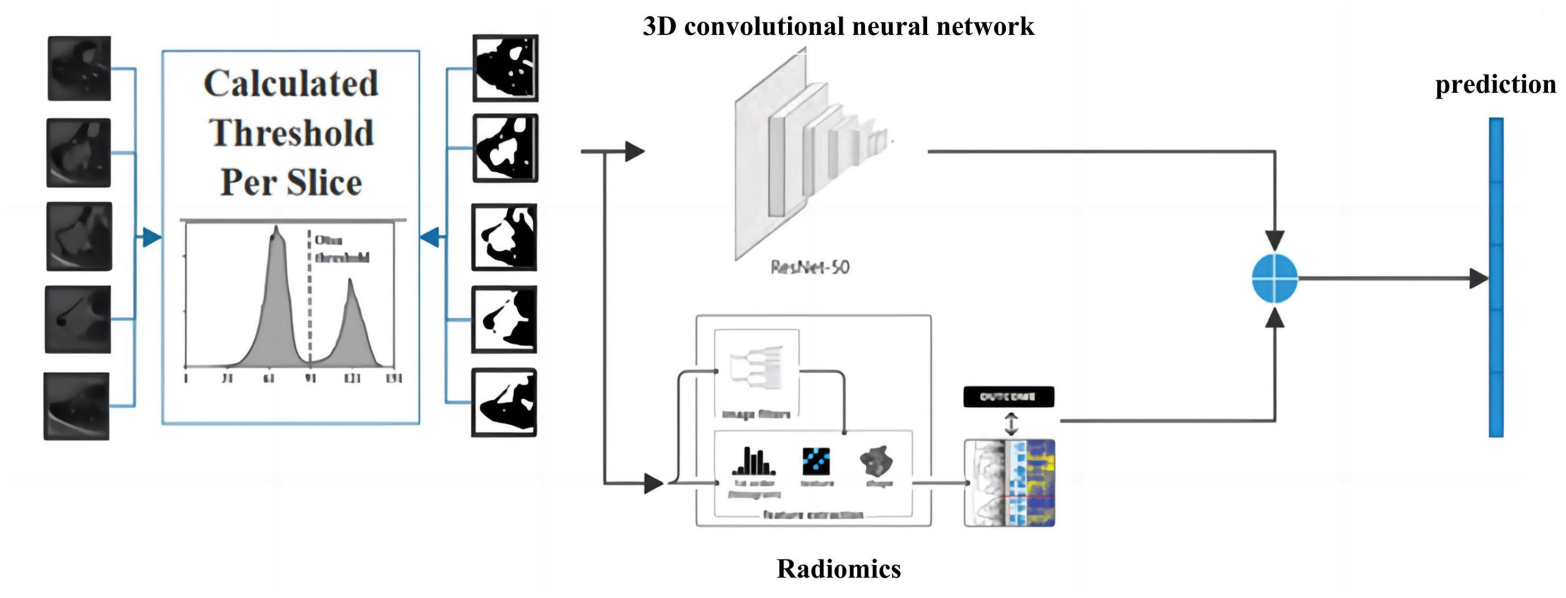
The architecture of the proposed model.

To better utilize the information of small pulmonary solid nodules in three-dimensional space, this paper combines 3D residual convolutional neural networks and radiomics to achieve nodule classification using complementary information between different modalities. As shown in Figure 7, the 3D visual and radiomic features information of small pulmonary solid nodules are input to 3D residual convolutional neural networks and multilayer perceptron, respectively. The two features are fused, and the results are output through the prediction layer.

**Figure 8:**
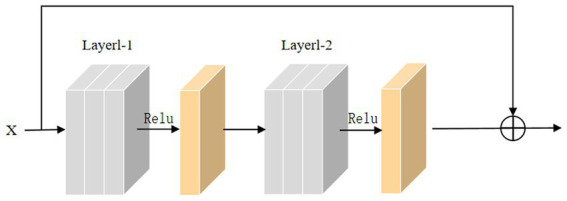
Diagram of skip connection module.

In the training process, the backpropagation of gradients is usually affected by the depth of the network, and more layers easily cause poor training results. He et al. introduced skip connections on convolutional neural networks to alleviate the gradient disappearance due to the over-deepening of the network. Inspired by this structure, this paper adds a skip connection structure to the 3D convolutional module, and the 3D skip connection module is shown in Figure 8. The equation of this module can be defined as:


(8) $$R\left(x\right)=W_{l}\left(f\left(W_{l-1}\left(x\right)\right)\right)$$

(9) U(x)=R(x)+M(x)

where *x* is the input to the skip connection module, *M*(*x*) is the skip connection, *U*(*x*) is the original function, and *R*(*x*) is the residual function.

In the 3D convolution operation, the input is convolved by the 3D convolution kernel, and bias is added. The 3D feature map is output using the normalization layer and the nonlinear activation unit. The formula for 3D convolution operation is as follows:


(10) $$\text{out}\left(N_{i},C_{{\text{out}}_{j}}\right)=\text{bias}\left(C_{{\text{out}}_{j}}+\sum_{k=0}^{C_{\text{in}}-1}\text{weight}\left(C_{{\text{out}}_{j}},k\right)*\text{input}\left(N_{i},\;\;k\right)\right)$$

where *N*, *C*, *D*, *H*, and *W* represent the batch size, number of channels, number of slices, length of slices, and width of slices, respectively. The operators ? are 3D interpolation operations. Meanwhile, Rectified Linear Unit (ReLU) and 3D BatchNorm are used in the model.

The residual convolutional neural network is designed based on the traditional convolutional neural network, which uses multiply subsampled to expand the local receptive field. However, the multiple subsampled operations will lead to a severe loss of small nodule features in the small solid pulmonary nodule classification task. This paper uses radiomics to solve the problem of information loss caused by subsampled. Radiomic features have correlation and complementarity with visual features. Specifically, the fusion of radiomic and visual features can complement the lost information from the statistical dimension of shape representation, making the network more robust. In addition, this paper uses dimensionality reduction to keep the visual features and radiomic features in the same dimension, and the two features are fused and input to the classifier to generate predictions. The detailed structure of the proposed network model is shown in Table 1.

**Table 1:** The overall structure of the proposed model.

Layer name	Output size	3D-ResNET50	Radiomics
Conv_1	128 × 128 × 128	7 × 7, 64, stride 2	/
		3 × 3 max pool, stride 2	/
Layer_1	56 × 56 × 56	$\left[\begin{array}{c} 1x1x1,64\\ 3x3x3,64\\ 1x1x1,256 \end{array}\right]x3$	/
Layer_2	28 × 28 × 28	$\left[\begin{array}{c} 1x1x1,128\\ 3x3x3,128\\ 1x1x1,512 \end{array}\right]x4$	/
Layer_3	14 × 14 × 14	$\left[\begin{array}{c} 1x1x1,256\\ 3x3x3,256\\ 1x1x1,1024 \end{array}\right]x6$	
Layer_4	7 × 7 × 7	$\left[\begin{array}{c} 1x1x1,512\\ 3x3x3,512\\ 1x1x1,2048 \end{array}\right]x3$	
	1 × 1 × 1	Average pool	107-d
Layer_Linear		Linear(256-d)	
Layer_MLP			MLP (256-d)
Layer_concat	512-d	Concat (Layer_Linear, Layer_MLP)	

### 2.3. Training and prediction

The training of the model can be divided into three steps. First, we follow the steps in the previous section to extract the cube containing the nodule area and obtain the mask of the nodule tissue by the Otsu thresholding algorithm. The mask can filter out the useless regions in the cube and also serve as the annotation for the radiomics extraction; Second, we resample the processed cube and expand the training data by the data enhancement methods, such as random flip and random radiation; Third, the enhanced cubes and the corresponding radiomics are input into the network for training.

After training, the data in the test set can perform nodule classification with the saved weights. We first extract the cube containing the nodules and obtain the optimal threshold by the Otsu thresholding algorithm. Then the extracted nodules are processed as in the training stage to obtain the radiomic features and the filtered nodule cube. Finally, the two groups of features are input to the trained network model to get the results.

## 3. Experimental results and analysis

The Adam optimizer trains the model with momentum set to ${\text{beta}}_{1}=0.9,{\text{beta}}_{2}=0.999$. We train 100 iterations, with the initial learning rate set to 0.001 and the learning rate dropping by 10% every ten iterations. In addition, dropout is set to 0.5, the batch size is set to 16, and the loss function is binary cross-entropy.

The datasets in the experiments came from the cooperative hospitals, which are non-public datasets, and the training set and the validation set have 1,429 samples in total. These samples are randomly divided into five subsets, represented as ${\text{subset}}_{0},{\text{subset}}_{1},{\text{subset}}_{2},{\text{subset}}_{3},{\text{subset}}_{4}$. One of them is taken as the validation set for each experiment, while the other subsets are used as the training set. In addition, 200 additional samples were collected as the test set to verify the robustness of the model and to ensure its effectiveness in practice, and these data were collected from different hospitals in independent time and independent devices.

The experiments mainly use classification accuracy, receiver operating characteristic (ROC) curve, and area under curve (AUC) values to analyze and evaluate the results. The accuracy is an indicator that can directly judge the inference ability of the model and can be defined as follows:


$$\text{Accuracy}=\frac{\text{TP}+\text{TN}}{\text{TP}+\text{TN}+\text{FP}+\text{FN}}$$

where TP, TN, FN, and FP are the numbers of true positive, true negative, false negative, and false positive pixels, respectively. Considering the influence of the optimal threshold in the task of small pulmonary solid nodules, we introduced ROC into the evaluation index and plotted ROC curves based on the false positive rate (FPR) and true positive rate (TPR) of the predicted results.


$$\text{TPR}=\frac{\text{TP}}{\text{TP}+\text{FN}},\left(0\leq \text{TPR}\leq 1\right).$$

$$\text{FPR}=\frac{\text{FP}}{\text{FP}+\text{TN}},\;\;\left(0\leq \text{FPR}\leq 1\right).$$

AUC is the area covered by the ROC curve, and the value can visually reflect the good or bad performance of the classifier under different thresholds, which range from 0 to 1. The higher the ACU value, the better the classifier performance.

**Figure 9:**
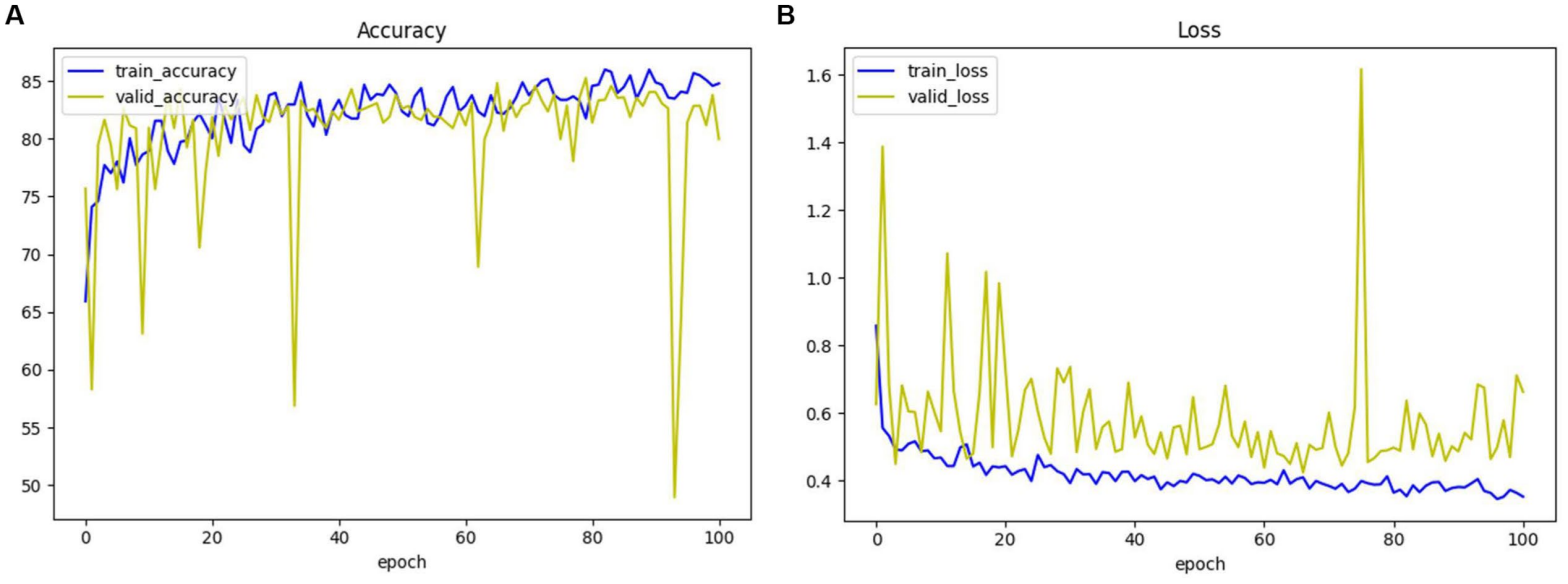
The accuracy and loss of 3D ResNet + Radiomics network in training and validation. **(A)** Loss. **(B)** Accuracy.

For the convenience of expressing the model, we will use 3D ResNet to denote the three-dimensional residual convolutional neural network, 3D VGG to denote the three-dimensional VGG model, and 3D ResNext to represent the three-dimensional ResNeXt model in the following. The accuracy and loss of the 3D ResNet + Radiomics network in training and validation are presented in Figure 9, and these values can be used as a basis for model convergence when they tend to be stable.

**Figure 10:**
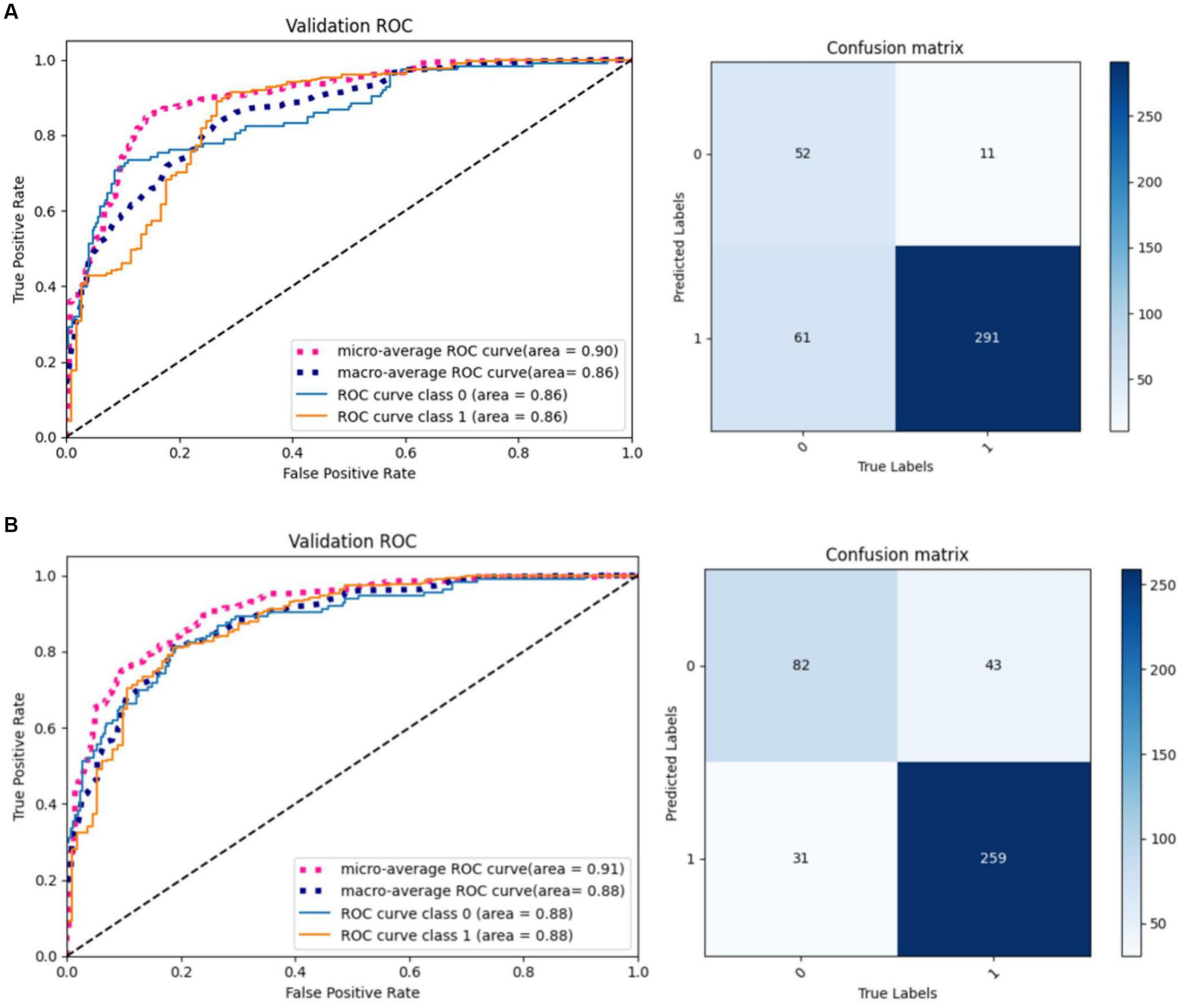
Results of the proposed model in two training. **(A)** Result 1. **(B)** Result 2.

The same model may find different local optimal solutions during two training processes, which causes differences in model performance. This paper selects the model weights that perform best on the validation set for testing. As shown in Figure 10, the same model under different training achieves different results on the validation set, and it can be observed that the model performance of result 2 is better than result 1.

### Ablation experiments of radiomics

**Figure 11:**
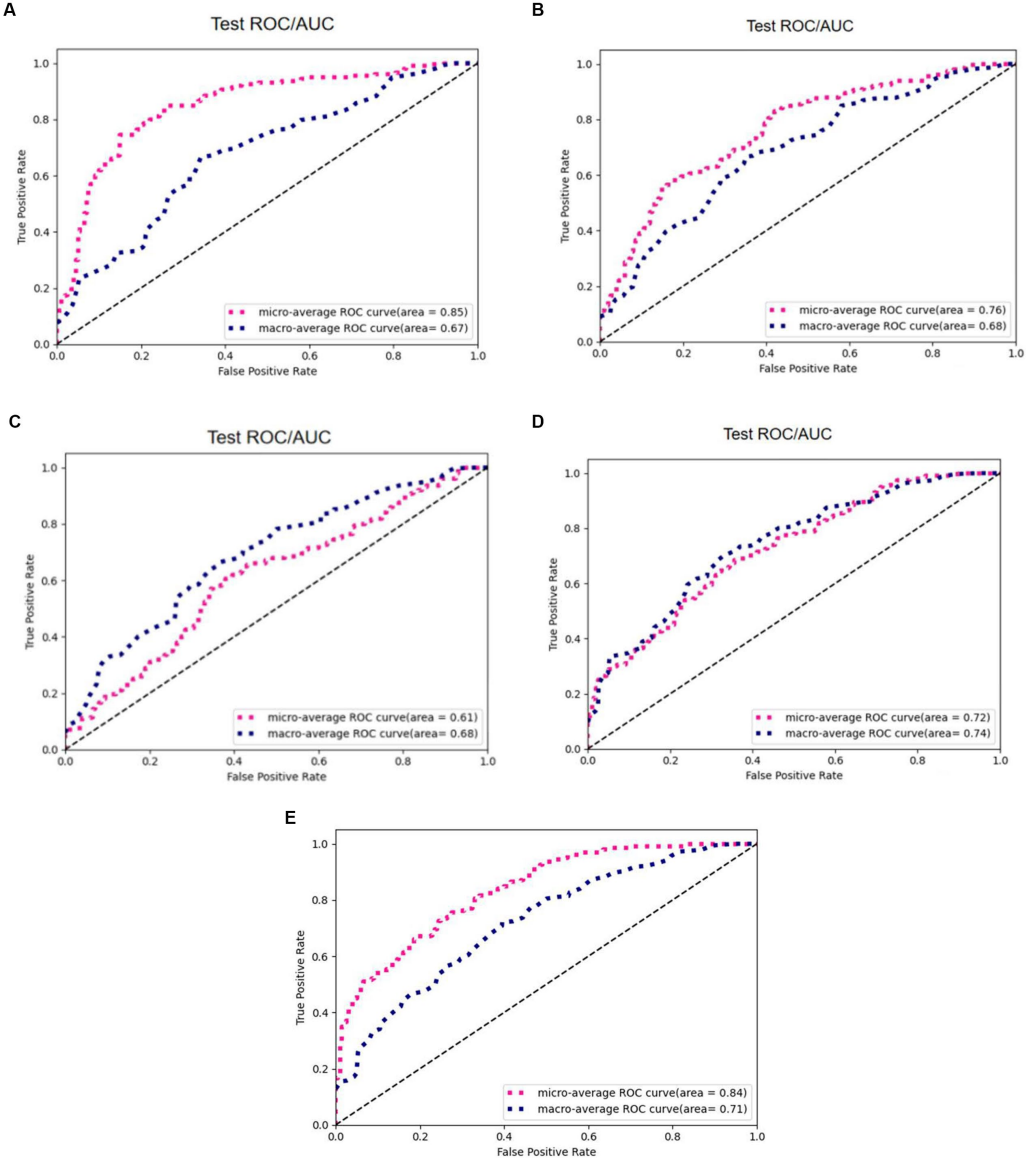
AUC/ROC of the models on the test set. **(A)** 3D ResNet50. **(B)** 3D ResNext. **(C)** 3D VGG. **(D)** 3D DenseNet. **(E)** 3D ResNet50+Radiomics.

To verify the effectiveness of radiomics for the classification task, we compared five different models, including 3D ResNet, 3D ResNeXt, 3D VGG, 3D DenseNet, and the proposed 3D ResNet + Radiomics model, and these experiments used the same preprocessing. Figure 11 reports the AUC/ROC of different models on the same test set, and the proposed model obtained better results. Radiomics alleviate the effect of information loss caused by downsampling in the convolutional neural network, which enables the model to learn more features and show more robustness on the test set.

### Comparison experiments between the Otsu thresholding algorithm and manual thresholding algorithm

**Figure 12:**
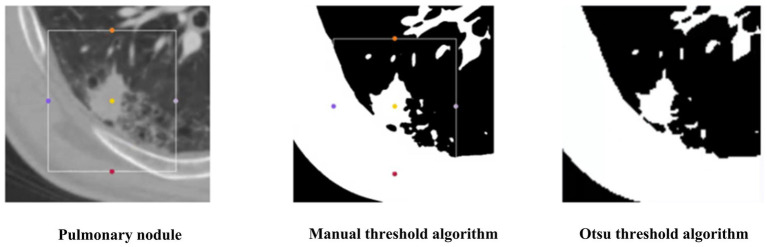
The segmentation results of two threshold algorithms.

To verify the superiority of the Otsu thresholding algorithm for preprocessing, we compared the Otsu thresholding algorithm and the manual thresholding algorithm. Figure 12 shows the visualization results of both algorithms on the same pulmonary nodules. The manual thresholding algorithm sets the range of HU values of CT images from 0 to 300, which is referenced to the common range of human tissues and experimental results and is more sensitive to solid tissues and lung cavities.

**Figure 13:**
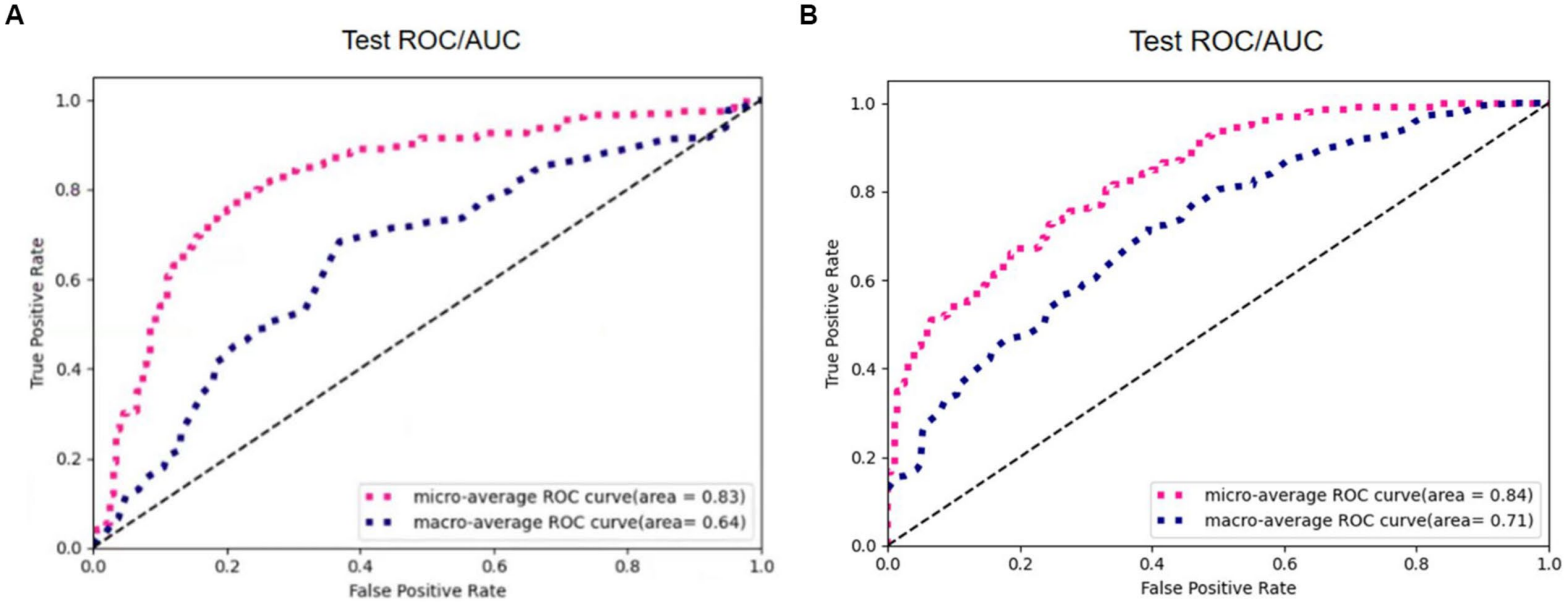
The results of 3D Resne50 + Radiomics with different threshold algorithms. **(A)** Manual thresholding algorithms. **(B)** Otsu thresholding algorithms.

The experimental results of the two algorithms are shown in Figure 13. The Otsu thresholding algorithm filters the tissue information around the small solid pulmonary nodules and achieves better results. On the one hand, the Otsu thresholding algorithm can provide flexible threshold adjustment to prevent filtering out the valuable information of nodules. On the other hand, the manually delineated thresholds lack the necessary flexibility in practical application and perform unstably on CT data facing different devices and batches, making the process of filtering information risky and unsuitable for application in practice.

Table 2 reports the results of multiple algorithms under different conditions, and the proposed algorithm achieves the best results. Compared with 3D ResNet50, 3D ResNet50 + Radiomics has a more significant improvement in Macro-Average AUC while keeping Micro-Average AUC unchanged.

**Table 2:** Comparison between different methods with and without our refinement.

Method	M	O	R	Micro-Average AUC	Macro-Average AUC
3D Resnet50	×	√	×	85%	67%
3D ResNext	×	√	×	76%	68%
3D DenseNet	×	√	×	72%	74%
3D VGG11	×	√	×	61%	68%
3D Resne50 + Radiomics	√	×	√	83%	64%
3D Resne50 + Radiomics (ours)	×	√	√	84%	71%

## 4. Conclusion

In this work, we propose a typing method based on the Otsu thresholding algorithm for small pulmonary solid nodules. The Otsu thresholding algorithm filters the tissue information around the small pulmonary solid nodules and reduces the interference of useless information. In addition, 3D visual and radiomic features are integrated to prevent missing features, and extensive experiments demonstrate the feasibility and interoperability of the two methods.

## Data availability statement

The raw data supporting the conclusions of this article will be made available by the authors, without undue reservation.

## Author contributions

MM proposed the main idea and did all the experiments. ZY contributed to part of the idea and experiments’ analysis. YZ improved the idea and provided datasets and experimental evaluation. All authors contributed to the article and approved the submitted version.

## Funding

Ph. D. Startup Fund of Hubei University of Technology.

## Conflict of interest

The authors declare that the research was conducted in the absence of any commercial or financial relationships that could be construed as a potential conflict of interest.

## Publisher’s note

All claims expressed in this article are solely those of the authors and do not necessarily represent those of their affiliated organizations, or those of the publisher, the editors and the reviewers. Any product that may be evaluated in this article, or claim that may be made by its manufacturer, is not guaranteed or endorsed by the publisher.
